# Shenhuang Granules for patients with sepsis: study protocol of a multicenter, randomized, double-blind, placebo-controlled clinical trial

**DOI:** 10.3389/fmed.2025.1700749

**Published:** 2025-11-21

**Authors:** Meng-Yuan Shen, Ze-Jiong Li, Dan-Dan Feng, Dong-Dong Yang, Shan Liu, Yi-Heng Fu, Bang-Jiang Fang, Jian-Nong Wu

**Affiliations:** 1 The First Affiliated Hospital of Zhejiang Chinese Medical University (Zhejiang Provincial Hospital of Chinese Medicine), Hangzhou, Zhejiang, China; 2 Department of Intensive Care Unit, The First Affiliated Hospital of Zhejiang Chinese Medical University (Zhejiang Provincial Hospital of Chinese Medicine), Hangzhou, Zhejiang, China; 3 Department of Emergency, The First Affiliated Hospital of Zhejiang Chinese Medical University (Zhejiang Provincial Hospital of Chinese Medicine), Hangzhou, Zhejiang, China; 4 Center of Clinical Evaluation, The First Affiliated Hospital of Zhejiang Chinese Medical University (Zhejiang Provincial Hospital of Chinese Medicine), Hangzhou, Zhejiang, China; 5 Department of Emergency, LongHua Hospital, Shanghai University of Traditional Chinese Medicine, Shanghai, China

**Keywords:** Shenhuang Granules, sepsis, randomized controlled trial, study protocol, Traditional Chinese Medicine

## Abstract

**Background:**

Sepsis is a life-threatening condition characterized by organ dysfunction due to a dysregulated host response to infection, with a persistently high mortality rate. In recent years, the role of Traditional Chinese Medicine in treating sepsis has gained recognition both domestically and internationally. Shenhuang Granules (SHG), composed of six ingredients: *Panax ginseng* C.A.Mey. (Ginseng Radix et Rhizoma), *Rheum palmatum* L. (Rhei Radix et Rhizoma), *Sargentodoxa cuneata* (Oliv.) Rehder & E.H.Wilson (Sargentodoxae Caulis), *Taraxacum mongolicum Hand.-Mazz*. (Taraxaci Herba), *Aconitum carmichaelii* Debeaux (Aconiti Lateralis Radix Praeparata), and *Whitmania pigra* (Whitman, 1884) (Hirudo), has been reported to strengthen and consolidate vital energy, clear heat and detoxify, promote qi circulation and gastrointestinal function, and enhance blood circulation. This study aims to evaluate the clinical efficacy and safety of SHG in patients with sepsis.

**Methods and analysis:**

A multicenter, randomized, double-blind, placebo-controlled clinical trial will be conducted and will enroll 410 patients aged ≥ 18 years who meet the diagnostic criteria of Sepsis 3.0. Participants will be randomly assigned in a 1:1 ratio to either the SHG group or the placebo group via a central randomization system. The SHG group will receive 7 days of standard sepsis bundle management combined with SHG, while the placebo group will receive the same management with a placebo for 7 days. Each dose will be dissolved in 100 mL of warm water (approximately 40 °C) and administered twice daily either orally or via a feeding tube (gastric or intestinal). The primary endpoint was 28-day all-cause mortality. Secondary endpoints include 28-day cumulative mechanical ventilation-free days, APACHE II score, SOFA score, overall mortality rate, total hospital stay, hospitalization cost, and inflammatory factors. Adverse events will be recorded throughout the study period.

**Discussion:**

This trial represents the first multi-center randomized controlled study in China evaluating the effect of SHG on patients with sepsis. The findings are expected to provide robust evidence regarding the efficacy and safety of SHG in sepsis management, offering evidence-based recommendations for clinical practice.

**Clinical trial registration:**

## 1 Introduction

Sepsis is a syndrome marked by a dysregulated host response to infection, leading to life-threatening organ dysfunction (1). Globally, an estimated 19 million cases of sepsis and approximately 5 million sepsis-related deaths occur annually (2). Epidemiological data from China show that over 20% of ICU patients have sepsis, with a 28-day mortality rate exceeding 30%, making it the leading cause of ICU deaths (3). As such, sepsis posed a significant threat to patient health, placed a substantial burden on healthcare systems, and remained a major challenge for the medical community.

Currently, sepsis treatment primarily relies on early anti-infective therapy, fluid resuscitation, vasoactive drugs, and organ support (4). However, the emergence of drug-resistant pathogens limits therapeutic options. Moreover, the systemic inflammatory response syndrome induced by sepsis is highly complex (5). A lack of specific anti-inflammatory treatments makes it difficult to mitigate the damage from excessive inflammation, posing serious obstacles to effective therapy. Therefore, novel and safer treatment strategies are urgently needed.

Traditional Chinese Medicine (TCM), as an important part of complementary and alternative medicine, has been widely applied in the prevention and treatment of severe diseases. According to TCM theory, sepsis falls under the category of “warm heat disease.” It refers to the pathological state with a rapid consumption of the vital Qi in the human body, caused by acute and severe pathological factors such as hemorrhage, loss of fluid, and all kinds of trauma. These corresponded with the pathophysiological understanding of sepsis in Western medicine (6). Based on these principles, our team proposed an early “truncation and reversal” treatment strategy. This involves using compound formulations of Chinese material medica early in the disease course to eliminate pathogenic toxins and stabilize yin, thereby halting sepsis progression. We developed SHG, a compound of six ingredients: *Panax ginseng* C.A.Mey. (Ginseng Radix et Rhizoma), *Rheum palmatum* L. (Rhei Radix et Rhizoma), *Sargentodoxa cuneata* (Oliv.) Rehder & E.H.Wilson (Sargentodoxae Caulis), *Taraxacum mongolicum* Hand.-Mazz. (Taraxaci Herba), *Aconitum carmichaelii* Debeaux (Aconiti Lateralis Radix Praeparata), and *Whitmania pigra* (Whitman, 1884) (Hirudo) (7). SHG has been used in our center to treat sepsis patients. Prior studies have showed that SHG improves recovery in critical COVID-19 patients, appears to prevent disease progression to critical stages (8). However, the efficacy and safety of SHG in sepsis caused by bacterial and other non-viral infections remain unverified. Therefore, we aim to explore the efficacy and safety of SHG in sepsis treatment in a multicenter, double-blind, randomized controlled trial (RCT).

## 2 Methods and analysis

### 2.1 Study design

**Figure 1:**
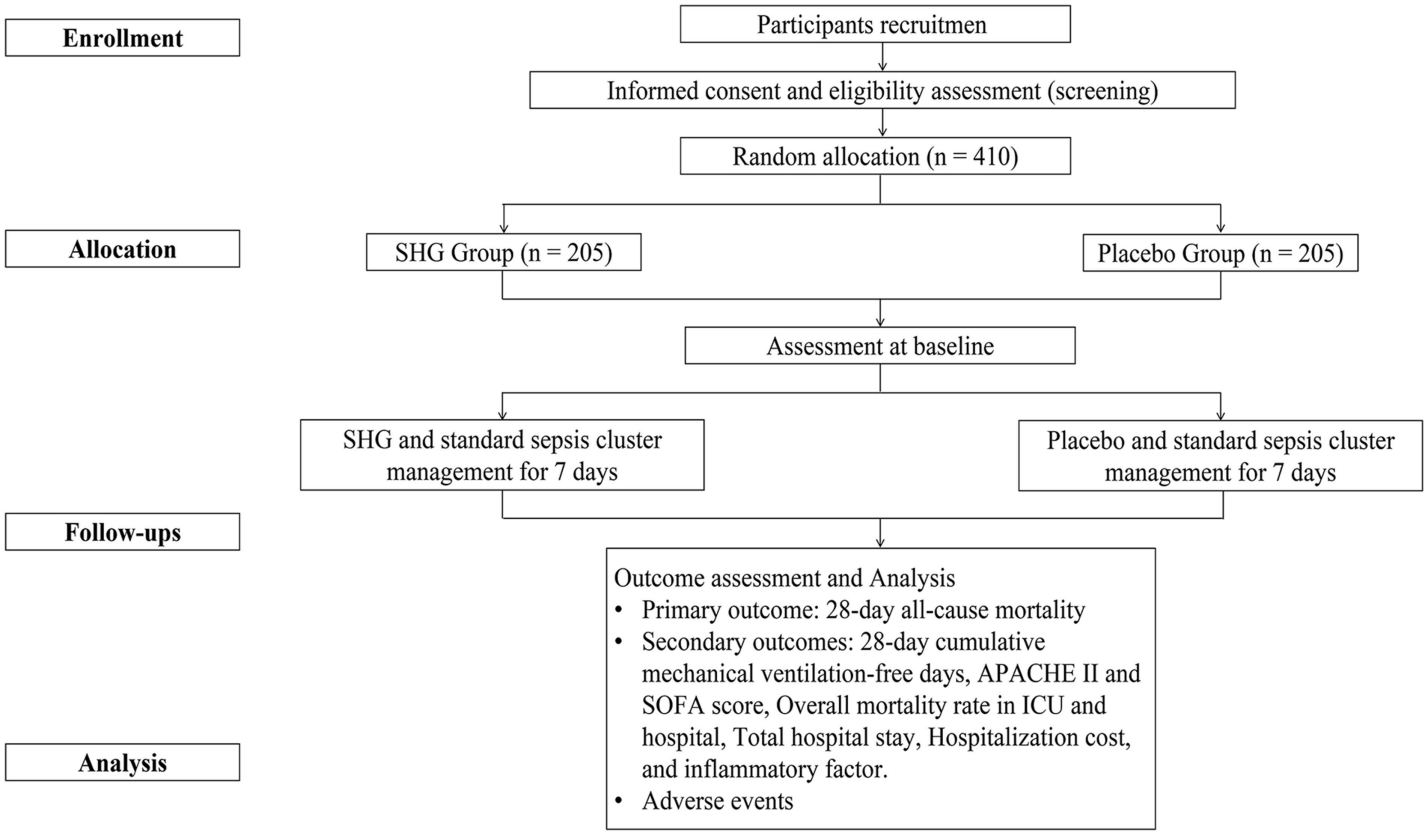
Flowchart of this study.

This multicenter, double-blind, parallel-group, placebo-controlled RCT is being conducted at the First Affiliated Hospital of Zhejiang Chinese Medical University, the First Affiliated Hospital of Zhejiang University School of Medicine, Hangzhou Red Cross Hospital, and Ningbo Hospital of Traditional Chinese Medicine. 410 patients will be enrolled, with 110 patients recruited from the lead site—the First Affiliated Hospital of Zhejiang Chinese Medical University—and 100 patients from each of the other three centers. Recruitment is scheduled from May 1, 2025, to December 1, 2026. The intervention period will last 7 days, and follow-up will continue for up to 28 days. Study visits will be conducted at baseline and on Days 7, 14, and 28. All participants will be required to sign written informed consent before randomization. This protocol adheres to the Standard Protocol Items: Recommendations for Interventional Trials (SPIRIT) guidelines (Supplementary material 1) (9). The study flow diagram is shown in Figure 1. Trial process chart is shown in Table 1.

**Table 1:** Trial process chart.

Study period	Enrolment	Day 0 of treatment	Day 7 of treatment	Day 14 of treatment	Day 28 of treatment	Close-out
						Discharged
Patient enrolment						
Signing informed consent	×					
Eligibility criteria	×					
Baseline characteristics	×					
Randomization/allocation	×					
Primary outcome						
All-cause mortality 28 days					×	
Secondary outcomes						
28-day cumulative mechanical ventilation-free days					×	
APACHE II score		×	×	×		
SOFA score		×	×	×		
Overall mortality rate						
Overall mortality rate (ICU)						×
Overall mortality rate (hospital)						×
Total hospital stay						
Total hospital stay (ICU)						×
Total hospital stay (hospital)						×
Hospitalization cost						
Hospitalization cost (ICU)						×
Hospitalization cost (hospital)						×
ESR, CRP, IL-6, IL-10, TNF-α, neutrophil percentage, and PCT		×	×	×		
Blood routine		×	×	×		
Urine routine		×	×	×		
Stool routine		×	×	×		
Liver and kidney function		×	×	×		
AEs		×	×	×	×	

### 2.2 Eligibility criteria

A total of 410 participants will be recruited across four hospitals. Critical care specialists will screen potential participants and assess eligibility based on the inclusion criteria. A research assistant will provide informed consent forms to eligible patients. The informed consent form includes a dedicated section that explicitly details the collection, long-term storage, and use of anonymized participant data and biological specimens. Participants are specifically informed that their data and specimens may be used for both the pre-specified biomarker analysis in this trial and for future ethically approved research related to sepsis and its mechanisms. The form clarifies that all samples will be coded and stored securely, and that participants retain the right to refuse data or specimen use for future studies without any impact on their medical care or standing in the current clinical trial.

### 2.3 Inclusion and exclusion criteria

Patients who meet the following criteria will be enrolled: (1) Age ≥ 18 years; (2) Meet the diagnostic criteria of Sepsis 3.0 guidelines (10); (3) Agree to participate and sign the informed consent form.

Patients who meet any of the following conditions will be excluded: (1) A Sequential Organ Failure Assessment (SOFA) subscore ≥ 3 for either liver or kidney function (11); (2) Expected death within 48 h, SOFA ≥ 13, or refusal of active treatment; (3) Known allergy or hypersensitivity to the study drugs; (4) Have received chemotherapy, radiotherapy, or high-dose immunosuppressants within the past month; (5) Agranulocytosis (absolute neutrophil count < 0.5 × 10^9^/L); (6) Intestinal fistula with gastric retention, severe diarrhea (watery stool ≥ 3 times/day), or the absence of a distal nutritional tube; (7) Gastrointestinal bleeding, intestinal obstruction, or severely elevated intra-abdominal pressure (IAP ≥ 20 mmHg) requiring transfusion; (8) Participation in other clinical trials concurrently or within 30 days prior to randomization; (9) Pregnant or lactating women.

To ensure patient safety and trial validity, we excluded patients with hepatic/renal SOFA score ≥ 3, indicating organ failure linked to higher mortality (12, 13), and those with total SOFA ≥ 13, a threshold predicting > 80% in-hospital mortality (14). These criteria reduce baseline heterogeneity and safety risks from altered drug metabolism in organ failure, while avoiding enrollment of patients with near-futile prognosis, thus preserving ethical equipoise and outcome interpretability. Although generalizability to the most severe cases is limited, this approach is appropriate for this initial SHG trial. We will report screening logs to support future studies in advanced organ failure populations.

### 2.4 Sample estimation

The sample estimation based on a related study (15), in which the 28-day mortality was 35.3% in the experimental group versus 48.3% in the control group. PASS 17.0 software was used to calculate the required sample size for comparing two proportions, with a significance level (*α*) of 0.05 and statistical power (1– *β*) of 0.8. A 1:1 allocation ratio required at least 175 patients per group. Considering a 15% dropout rate, at least 205 patients were needed per group, totaling a minimum of 410 participants.

### 2.5 Randomization and blinding

Block randomization will be used to assign eligible participants to the SHG or placebo group in a 1:1 ratio. Although formal stratification factors are not used in the randomization process, key prognostic factors—including study site, baseline disease severity, and infection type—will be pre-specified as adjustment variables in the statistical analysis to control for potential imbalances. The block size is 4. A central randomization system will manage both allocation and drug distribution. SHG and placebo, packaged and labeled blindly by Tianjin Hongri Pharmaceutical Co., Ltd., will be distributed to trial sites. Each treatment assignment (active drug or placebo) will be linked to a unique random number and corresponding drug number, establishing a one-to-one match with each subject. From randomization to database lock, all individuals involved-including participants, investigators, data analysts, sponsors, and medical staff—will remain blinded to the treatment assignments.

### 2.6 Emergency unblinding

Under normal conditions, the study will remain blinded until all participants completed the treatment phase and the trial database was finalized. Emergency unblinding will be permitted only in critical situations. In cases of serious adverse events (SAEs), knowledge of the participant’s treatment assignment may be necessary for appropriate clinical management. In such instances, the site’s responsible investigator and the principal investigator will jointly decide whether to proceed with unblinding. If unblinding was deemed necessary, the project leader will be notified in advance, and the central investigator unblinded the participant via the system. Once unblinded, the participant will be immediately withdrawn from the treatment phase.

### 2.7 Intervention

Patients in the experimental group will receive SHG for 7 days (16), while those in the control group will receive placebo granules for 7 days. Both groups will continue to receive sepsis bundle care according to the Surviving Sepsis Campaign: Guidelines for the Management of Critically Ill Adults with Sepsis and Septic Shock–2021 Update.

Each SHG package contained the following components:

1. 15 g *Panax ginseng* C.A.Mey. (Araliaceae)—Ginseng Radix et Rhizoma (dried root and rhizome).
2. 9 g *Rheum palmatum* L. (Polygonaceae)—Rhei Radix et Rhizoma (dried root and rhizome).
3. 15 g *Sargentodoxa cuneata* (Oliv.) Rehder & E.H.Wilson (Lardizabalaceae)—Sargentodoxae Caulis (dried stem).
4. 15 g *Taraxacum mongolicum* Hand.-Mazz. (Asteraceae)—Taraxaci Herba (whole plant).
5. 9 g *Aconitum carmichaelii* Debeaux (Ranunculaceae)—Aconiti Lateralis Radix Praeparata (processed lateral root).
6. 3 g *Whitmania pigra* (Whitman, 1884) (Annelida; Hirudinea)—Hirudo (dried whole body of leech).

Botanical identities of plant materials were verified according to Plants of the World Online (POWO, Royal Botanic Gardens, Kew), and the animal species *W. pigra* was validated via NCBI Taxonomy (TaxID 486152). The *W. pigra* medicinal material used in this study is acquired in accordance with The Chinese Pharmacopoeia (2020 Edition) (17). The material comes from an artificially bred population, not a wild catch. All components are sourced from licensed Chinese medicinal material suppliers, and none originate from or contain endangered species.

Placebo granules consist of 98% maltodextrin and 2% SHG to match SHG in odor, color, taste, and texture. The components of SHG are shown in Table 2.

**Table 2:** Components of Shenhuang Granules (SHG): botanical and pharmacopoeial identities.

Scientific name (italicized)	Pharmacopoeial name (non-italic)	Dosage (g)	Part used/processing
*Panax ginseng* C.A.Mey. (Araliaceae)	Ginseng Radix et Rhizoma	15	dried root and rhizome
*Rheum palmatum* L. (Polygonaceae)	Rhei Radix et Rhizoma	9	dried rhizome/root
*Sargentodoxa cuneata* (Oliv.) Rehder & E.H.Wilson (Lardizabalaceae)	Sargentodoxae Caulis	15	dried stem
*Taraxacum mongolicum* Hand.-Mazz. (Asteraceae)	Taraxaci Herba	15	whole plant
*Aconitum carmichaelii* Debeaux (Ranunculaceae)	Aconiti Lateralis Radix Praeparata	9	processed lateral root
*Whitmania pigra* (Whitman, 1884) (Annelida; Hirudinea)	Hirudo (Shuizhi)	3	dried whole body of leech

Botanical names validated in POWO (Royal Botanic Gardens, Kew); animal species (*Whitmania pigra*) verified via NCBI Taxonomy (TaxID 486152).

Pharmacopoeial names follow Chinese Pharmacopoeia (2020 Edition).

Italicize genus and species only; authors and family names are non-italic.

Both SHG and placebo are provided by Tianjin Hongri Pharmaceutical Co., Ltd. Each dose will be dissolved in 100 mL of warm water (approximately 40 °C), administered twice daily either orally or via a feeding tube (gastric or intestinal).

**Figure 2:**
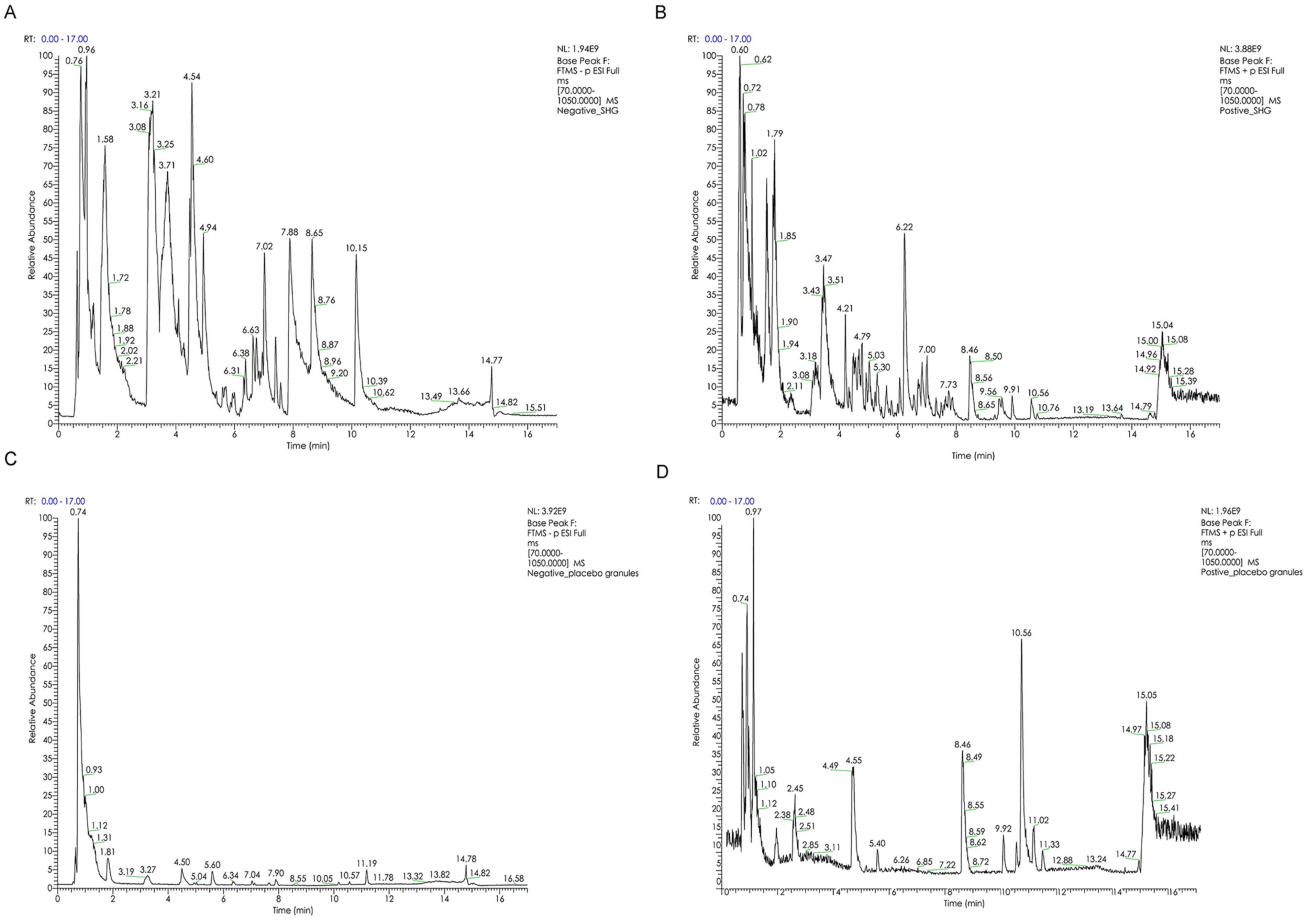
**(A)** BPI chromatogram of SHG in negative ion mode. **(B)** BPI chromatogram of SHG in positive ion mode. **(C)** BPI chromatogram of placebo granules in negative ion mode. **(D)** BPI chromatogram of placebo granules in positive ion mode.

The SHG composition is determined based on the general principles of The Chinese Pharmacopoeia (2020 Edition), using gas chromatography (General Principle 0512) (17). Quality control analysis is performed by Sanshu Biotechnology Co., Ltd., confirming that the product meets the Chinese medicine standards set by the State Food and Drug Administration. The certificate specimen is stored at the main experimental center—the Clinical Experiment Center of Zhejiang Hospital of Traditional Chinese Medicine. BPI chromatogram of SHG and placebo granules in different ion mode are available in Figure 2. Detailed methods and results regarding SHG and placebo quality control are available in Supplementary material 2. All medication information will be recorded in the case report form (CRF).

A designated caregiver will collect used and unused medication packages from the participant. An intensivist will provide counseling on sepsis management. Participants will be considered dropouts if they failed to complete the study for any reason or used prohibited medications.

To ensure consistent application and documentation of co-interventions (antibiotics, fluids, vasopressors, steroids) across all centers, all management will adhere to the Surviving Sepsis Campaign: International Guidelines for the Management of Sepsis and Septic Shock (2021). Prior to study initiation, all site investigators will receive mandatory, standardized training on these guidelines. Detailed co-intervention data—including antibiotic timing and selection, fluid volumes, and vasopressor type and maximum dose—will be systematically captured in the eCRF. An independent Data Monitoring Committee (DMC) will regularly audit protocol compliance and review aggregate data to identify and address any inter-center variations, thereby minimizing potential bias and enhancing trial validity.

Standardized Concomitant Care: All participating sites will implement guideline-concordant sepsis management, including early empiric antibiotics with de-escalation, crystalloid-based fluid resuscitation, norepinephrine as first-line vasopressor (target MAP ≥ 65 mmHg), source control, and organ support (lung-protective ventilation, RRT, ECMO). Adjunctive therapies comprise stress-ulcer/VTE prophylaxis (prophylactic-dose LMWH), analgesia/sedation, glycemic control (7.8–10.0 mmol/L), nutrition support, and electrolyte management. Corticosteroids are permitted for refractory septic shock with detailed documentation of indication, agent, dose and duration.

Prohibited Interventions and Documentation: During the 7-day intervention period, prohibited measures include: co-enrollment in other interventional trials; any systemic TCM formulations containing SHG-related constituents (*Aconiti Lateralis Radix Praeparata*, *Ginseng, Rhei Radix*, *Cistanche*, *Salvia miltiorrhiza*, or *Whitmania extracts*); and therapeutic-dose anticoagulation at randomization. All concomitant interventions will be systematically recorded using standardized eCRF fields capturing antibiotic timing/spectrum, fluid volumes (0–24/72 h), vasopressor dosing, corticosteroid use, source-control timing, VTE prophylaxis/therapy, and any protocol deviations with justification. The study drug may be temporarily withheld if prohibited therapies are clinically necessary, with such cases reviewed by the Safety Committee.

### 2.8 Primary outcome

The primary endpoint is all-cause mortality, measured from the date of randomization (Day 0) through Day 28. This refers to the proportion of patients who die from any cause within 28 days of sepsis diagnosis. For patients discharged alive from the ICU, outcome data will be obtained via telephone follow-up.

### 2.9 Secondary outcomes

The secondary outcomes include:

1. 28-day cumulative mechanical ventilation-free days: This refers to the total number of days a patient was able to breathe spontaneously without mechanical ventilation within 28 days within the first 28 days after randomization. This index reflects the recovery progress of respiratory function and was predictive of prognosis. A higher number indicates better recovery and therapeutic response, typically associated with higher survival rates and improved long-term outcomes. Conversely, a lower value suggests a poor treatment response, worse prognosis, and potentially increased mortality risk, necessitating adjustments to the treatment plan.
2. SOFA and acute physiology and chronic health evaluation II (APACHE II) scores (Days 0, 7, and 14): The SOFA score quantitatively assesses the extent and progression of organ dysfunction in ICU patients by monitoring six organ systems: respiratory, cardiovascular, hepatic, coagulation, renal, and neurological. Scores range from 0 (normal function) to 24 (most severe dysfunction) (12). The APACHE II score evaluates disease severity at ICU admission, factoring in physiological parameters, age, and chronic health status. A higher score may indicate a higher disease severity and a higher risk of death (18).
3. Overall mortality rate in ICU and hospital: This is defined as the proportion of all-cause deaths among ICU or hospitalized patients. Elevated mortality rates could indicate more severe illness, insufficient medical resources, or suboptimal clinical management.
4. Total hospital stay: Total hospital stay includes both ICU and general ward time (in days). ICU stay duration is measured from ICU admission to discharge (in hours or days). Shorter hospital stays generally reflect efficient treatment and resource use, while prolonged stays are associated with increased medical costs and greater financial burdens on patients and healthcare systems. Data will be collected at discharge.
5. Hospitalization cost: Hospitalization cost includes both ICU and general ward (in days). The reduction in ICU hospitalization costs may suggest that the drug accelerates patient recovery, decreases reliance on specialized ICU interventions, shortens ICU stays, and consequently lowers overall care expenses. Total hospitalization cost encompasses all expenses from admission to discharge, including ward fees, examination charges, medication costs, surgical fees, nursing fees, and more. This metric provides a comprehensive overview of the medical resources consumed during sepsis treatment. Given the complexity of sepsis management, which involves multiple departments and diverse treatment modalities, total hospitalization cost effectively illustrates the impact of various drug regimens on overall healthcare expenditure. A decline in total hospitalization cost could stem from the drug’s ability to reduce complications, eliminate unnecessary treatments, enhance therapeutic efficiency, and alleviate economic burdens.
6. Inflammatory factors: C-reactive protein (CRP) and erythrocyte sedimentation rate (ESR) serve as critical outcome indicators, reflecting the inflammatory state of patients from distinct perspectives. CRP is an acute-phase reactant synthesized by the liver in response to inflammatory stimuli. During sepsis, pathogens such as bacteria and viruses invade the body, activating the immune system, triggering an inflammatory cascade, and causing a rapid elevation in CRP levels. ESR refers to the rate at which red blood cells settle under standardized conditions, with its increase primarily associated with systemic inflammation and tissue damage. In sepsis, inflammation alters the composition of plasma proteins, particularly increasing fibrinogen and globulin levels, which promote the formation of red blood cell rouleaux and consequently accelerate erythrocyte sedimentation, leading to elevated ESR values. In addition, we will measure interleukin-6 (IL-6), IL-10, tumor necrosis factor-α (TNF-α), and procalcitonin (PCT) to more comprehensively capture sepsis-related inflammatory activation. IL-6 and TNF-α are central pro-inflammatory mediators in the sepsis cascade: circulating IL-6 correlates with disease severity and treatment response (19), while TNF-α drives early immune activation and organ injury (20). By contrast, IL-10 is a key anti-inflammatory cytokine that counterbalances these responses, with its level reflecting the patient’s immunoregulatory state (21). PCT is a relatively specific marker of bacterial infection, useful for distinguishing bacterial sepsis from non-bacterial inflammation and for tracking resolution (22). Finally, neutrophil percentage—a routine hematological index—provides an additional readout of neutrophil activation, an integral component of the septic immune response (23). Monitoring these inflammatory markers can aid in assessing treatment efficacy and predicting disease progression and prognosis.

### 2.10 Safety outcomes

During each research visit, the patients’ safety status is evaluated by assessing their vital signs, complete blood cell count, coagulation function, and gastrointestinal symptoms, such as nausea, vomiting, and diarrhea. Other evaluations include urine analysis, blood routine examination, liver and kidney function tests, and electrocardiogram results. All adverse events occurring during the research period will be closely monitored and meticulously recorded.

### 2.11 Adverse events

Given the known risks of *aconite* (cardiotoxicity/arrhythmia) and *W. pigra* (anticoagulation/bleeding), a proactive safety-monitoring strategy will be implemented (24, 25). All participants undergo a baseline and daily 12-lead ECG during the 7-day treatment, with continuous ICU telemetry. Serum potassium, magnesium, and high-sensitivity troponin will be measured at baseline, at least daily for electrolytes (with corrective targets K^+^ ≥ 4.0 mmol/L and Mg^2+^ ≥ 1.0 mmol/L), and on Day 3 and Day 7 for troponin. Drug discontinuation criteria include Mobitz II or third-degree AV block, sustained ventricular arrhythmia (≥30 s or requiring urgent intervention), QTcF ≥ 500 ms or an increase ≥ 60 ms from baseline, or symptomatic hypotension judged drug-related and not attributable to sepsis (26). For *Whitmania*-related bleeding risk, platelet count, PT/INR, aPTT, and fibrinogen will be assessed at the same timepoints. The study drug will be suspended if any of the following occur: platelet count < 50 × 10^9^/L, INR > 1.5, aPTT > 1.5 × laboratory control value, fibrinogen < 1.5 g/L, or major bleeding (per standard definitions). Prophylactic-dose anticoagulation is permitted in accordance with guidelines, whereas therapeutic anticoagulation at randomization is an exclusion criterion (27). If therapeutic anticoagulation becomes indicated after randomization (e.g., new VTE), standard care should proceed with temporary study-drug suspension; the patient remains in the ITT analysis set. SHG ingredients are considered safe at the recommended Chinese Pharmacopoeia (2020 Edition) dosages.

Interventions were conducted under the supervision of critical care specialists. Participants’ physical conditions and subjective reports are closely monitored. Safety outcomes included all adverse events (AEs) and SAEs observed during the study period. Clinically significant laboratory abnormalities were also recorded as AEs. All AEs were documented using a standardized reporting format and submitted to the academic lead. SAEs are reported to the Ethics Committee of Zhejiang Provincial Hospital of Traditional Chinese Medicine within 24 h.

A committee of medical experts determines whether adverse events are related to SHG. Zhejiang Provincial Hospital of Traditional Chinese Medicine bears the cost of treatment and provides compensation for any trial-related economic losses. Participants retain the right to withdraw from the study at any time for any reason. Any other unintended effects related to the intervention or study procedures are also documented and reported.

### 2.12 Data collection and management

All data will be recorded in CRFs and entered into an electronic database under the principal investigator’s supervision. An independent DMC will ensure the accuracy and completeness of data collection. Once data registration is completed, the database was secured to protect confidentiality. De-identified data sets will be forwarded to independent statisticians for final analysis.

The research team will conduct training sessions to familiarize staff with the study protocol. Each participating center will appoint dedicated quality inspectors, implement progress reporting systems, conduct regular evaluator training, and assess case quality periodically. An independent third-party unit will be responsible for monitoring clinical observations and data quality, and regularly audit project data.

At the main center, clinical evaluation will be carried out by staff not involved in patient treatment. Statistical analyses will be performed by this team to minimize bias in both the conduct and interpretation of the trial. Unified training across all centers will ensure consistent evaluation of efficacy indicators and improve the reliability of the results.

### 2.13 Quality control

All staff involved in the study are required to have Good Clinical Practice certification from the State Food and Drug Administration. Prior to study initiation, investigators will undergo comprehensive training and testing to ensure familiarity with the trial protocol and to improve compliance. Qualified researchers will be responsible for ensuring the accuracy and completeness of data entered into the CRFs, which will be securely maintained.

A designated quality control researcher will conduct monthly assessments to maintain high study quality. All monitoring procedures—including visit frequency and source data verification—will follow the standardized protocol.

### 2.14 Statistical analysis

Statistical analyses will be performed using SPSS version 27.0 (SPSS Inc., Chicago, IL, USA), with a two-tailed significance level of *p* < 0.05, following the intention-to-treat principle using the Full Analysis Set as the primary dataset and the Per-Protocol Set for supportive analyses. The primary outcome, 28-day all-cause mortality, will be analyzed using a multivariable logistic regression model adjusted for study site, baseline disease severity, and infection source. Missing data will be handled using multiple imputation under the missing-at-random assumption, supplemented by sensitivity analyses including worst-case scenario imputation. No formal adjustment for multiple comparisons will be applied to the primary outcome, while secondary outcomes will be interpreted with emphasis on effect sizes and their confidence intervals (CI).

Continuous variables will be summarized as mean (standard deviation) for normally distributed data or median (interquartile range) for non-normally distributed data, and analyzed using independent t-tests or Mann–Whitney U tests for between-group comparisons, with repeated-measures ANOVA or Wilcoxon signed-rank tests for longitudinal analyses. Categorical variables will be expressed as counts and percentages (%) and compared using the *χ*^2^ test or Fisher’s exact test, while ordinal data will be analyzed using non-parametric methods including the Wilcoxon rank-sum test.

To explore the consistency of the treatment effect, pre-specified subgroup analyses for the primary endpoint (28-day all-cause mortality) will be performed based on the following baseline factors: age, baseline disease severity, presence of septic shock, and source of infection. The treatment effect within each subgroup will be presented as a relative risk with its 95% CI. The statistical significance of subgroup differences will be tested by including a treatment-by-subgroup interaction term in the logistic regression model. A *p*-value for interaction of < 0.10 will be considered suggestive of a potential effect modification.

An independent DMC will conduct one interim analysis after 205 participants (50%) complete the 28-day follow-up. Safety will be assessed by comparing SAEs, with specific attention to *aconite*-related cardiotoxicity and *Whitmania*-associated bleeding; thresholds for action include an SAE rate in the SHG group that is double the placebo rate or exceeds 15%. Efficacy will be evaluated by analyzing 28-day all-cause mortality using a Lan–DeMets *α*-spending function with O’Brien–Fleming boundaries to control type I error. The DMC will report its recommendations to the Ethics Committee within 7 days, and no further interim analyses are planned if the trial continues.

## 3 Discussion

Sepsis has increasingly become a significant global health concern, with a notable rise in patient numbers in recent years (28). The persistence and severity of sepsis not only compromise patients’ quality of life but also place a substantial burden on society. Despite continuous updates to sepsis management guidelines, the mortality rate has not markedly decreased, and effective pharmacological treatments remain limited (29). The 2021 international guidelines for sepsis and septic shock management recommend early fluid resuscitation, infection control, and multi-organ supportive care. Additionally, hydrocortisone may be administered to patients with septic shock unresponsive to vasopressors (16). While glucocorticoids may shorten vasopressor use duration, concerns persist regarding their immunosuppressive effects and potential negative impact on long-term survival and quality of life. Therefore, the search for clinically effective and safe treatment alternatives remains urgent.

Growing evidence suggests that TCM can alleviate sepsis symptoms and improve patients’ quality of life (30, 31). The Chinese Sepsis/Septic Shock Emergency Treatment Guidelines recommend combining Shenfu injection with vasoactive drugs to help reduce mortality (32). In a multicenter RCT, Xuebijing injection significantly reduced 28-day all-cause mortality in patients with early-stage sepsis (33). The TCM approach to sepsis involves dual immunoregulatory effects—eliminating pathogenic factors while strengthening the body’s defenses.

SHG, a TCM compound composed of six medicinal ingredients—*P. ginseng* C.A.Mey. (Ginseng Radix et Rhizoma), *R. palmatum* L. (Rhei Radix et Rhizoma), *S. cuneata* (Oliv.) Rehder & E.H.Wilson (Sargentodoxae Caulis), *T. mongolicum* Hand.-Mazz. (Taraxaci Herba), *A. carmichaelii* Debeaux (Aconiti Lateralis Radix Praeparata), and *W. pigra* (Whitman, 1884) (Hirudo)—was developed according to this therapeutic rationale. Botanical nomenclature follows the standards of POWO and NCBI for taxonomic validation. Rhubarb is used as a purgative and bactericide to reduce internal heat, promote blood circulation, and resolve dampness. *Sargentodoxa cuneata* and *Taraxacum mongolicum* share properties of clearing heat, detoxification, blood activation, wind dispersal, and pain relief. *Panax ginseng* is believed to strongly tonify qi, nourish the lungs, promote salivation, quench thirst, and calm the mind. *Panax ginseng* can directly kill bacteria and modulate bacterial adhesion, inflammation, and cytotoxicity (34). Aconiti Lateralis Radix Praeparata is considered a stimulant for the spleen and kidneys and has shown antibacterial effects—especially against *S. aureus* and *E. coli*—as well as immunoregulatory functions (35, 36). *Whitmania pigra* is known for promoting blood circulation and resolving stasis. *Whitmania pigra* extract could effectively reduce deep vein thrombosis by modulating the SIRT1/NF-κB signaling pathway.

Beyond its traditional applications, a growing body of preclinical evidence supports the mechanistic basis for SHG in sepsis. Both the individual constituents and related formula preparations demonstrate multi-target activities pertinent to sepsis pathophysiology. *Panax ginseng* and its active ginsenosides (e.g., Rg1) have been shown to alleviate intestinal barrier disruption and suppress pro-inflammatory cytokines in CLP-induced sepsis, potentially through inhibition of the TLR4/NF-κB pathway, leading to reduced IL-6 and TNF-α levels (37). Similarly, *Salvia miltiorrhiza* constituents exert organ-protective effects: tanshinone IIA ameliorates sepsis-induced lung injury by downregulating ROCK2 and inactivating NF-κB (38), while salvianolic acid B attenuates hepatic and renal injury via antioxidant and anti-apoptotic mechanisms (39). Anthraquinones derived from Rhei Radix et Rhizoma, such as emodin and rhein, protect against lung and kidney injury in experimental sepsis models through SIRT1/HMGB1 and NF-κB pathways, respectively (40, 41). Furthermore, echinacoside from *Cistanche deserticola* mitigates lung injury and oxidative stress while improving endothelial function (42). *Whitmania pigra* (Whitman, 1884), a source of hirudin-related peptides, provides potent anticoagulant activity by inhibiting thrombin; recombinant hirudin analogs reduce coagulation activation and fibrin deposition in models of endotoxemia and disseminated intravascular coagulation (43, 44). Although direct preclinical studies of the complete SHG formulation are limited, related ginseng-aconite formulations such as Shenfu injection have demonstrated improved survival, intestinal mucosal protection, and reduced TNF-*α* in septic models (45). Moreover, network pharmacology analyses suggest that SHG itself modulates cytokine storm and inflammatory pathways in a multi-component manner (46). Collectively, these findings from reductionist constituent studies and emerging formula-level data provide a compelling mechanistic rationale for the clinical evaluation of SHG in sepsis.

Based on both TCM principles and modern pharmacological research, SHG appears to hold therapeutic potential for treating sepsis. Nevertheless, the safety of the six constituent ingredients must always be carefully considered. Particular attention is paid to *Aconiti Lateralis Radix Praeparata* (Fuzi) and *W. pigra* (Whitman, 1884) (Shuizhi). A proactive monitoring strategy is implemented: continuous ECG telemetry plus serial measurements of electrolytes and troponin (baseline, Days 3, 7) address Fuzi-related cardiotoxicity; concurrent platelet and coagulation tests manage Shuizhi-associated bleeding risk, with predefined suspension thresholds. Numerous preclinical and clinical studies have confirmed that the dosages used in SHG are well below toxic thresholds and are commonly employed in clinical settings with favorable outcomes (47–50). Furthermore, *W. pigra* is not included in the “National Key Protected Wild Animal List” or the Appendices of the Convention on International Trade in Endangered Species of Wild Fauna and Flora (CITES). Its artificial breeding technology is well-developed, and most of the animals in circulation are captive-bred (51, 52). The impact of usage on the overall population size is negligible.

In 2020, SHG was used in the treatment of COVID-19-related sepsis in Wuhan. Studies demonstrated that SHG significantly reduced mortality, improved prognosis, shortened hospitalization, and helped prevent disease progression (6). However, high-quality clinical data regarding its use in bacterial (non-COVID-19) sepsis remain limited. This trial aims to address this gap by evaluating the efficacy and safety of SHG in patients with bacterial sepsis.

Extraction and preparation of SHG are critical to its therapeutic effect. Our team has optimized the extraction process and implemented comprehensive quality control measures using a variety of evaluation indicators. In summary, this trial represents the first multicenter, double-blind, placebo-controlled RCT assessing the efficacy and safety of SHG in treating sepsis. The results are expected to provide high-quality clinical evidence supporting the use of SHG and expanding treatment options for patients with sepsis.

## 4 Limitations

This study has several limitations. First, the 7-day treatment and 28-day follow-up focus on short-term outcomes and may not capture delayed efficacy or late adverse events. Future studies should incorporate longer surveillance and patient-centered outcomes. Second, excluding patients with severe hepatic/renal dysfunction (SOFA subscore ≥ 3) and those with total SOFA ≥ 13 may limit generalizability to the sickest subgroup with advanced organ failure and near-futile short-term prognosis. In the future, the generalizability of research can be enhanced by conducting practical studies specifically for patients with severe liver and kidney dysfunction.

## 5 Conclusion

This study protocol outlines the design of a multicenter, randomized, double-blind, placebo-controlled trial to evaluate the efficacy and safety of SHG in patients with sepsis. The findings of this trial are expected to provide high-quality clinical evidence regarding the use of SHG as an adjunctive therapy for sepsis. If proven effective and safe, SHG could offer a valuable additional treatment option within the integrated management strategy for sepsis, potentially improving patient outcomes.
