# Supplementary material for: Shenhuang Granules for patients with sepsis: study protocol of a multicenter, randomized, double-blind, placebo-controlled clinical trial
Source: Front Med (Lausanne). 2025 Nov 21;12:1700749. doi: 10.3389/fmed.2025.1700749 (PMC12678277; doi:10.3389/fmed.2025.1700749)
Supplement: Supplementary file 2 [file Table_2.DOCX]

UPLC-Orbitrap-MS analysis of SHG and placebo granules

**1. Preparation of the SHG solution**

We weighed 100 mg of the SHG and put them into a centrifugation tube, subsequently adding 1 mL extraction solution (water/acetonitrile/isopropyl alcohol, 1:1:1), swirl them for 60 s, and extract them by ultrasound at low temperature for 30 min. The supernatant was centrifuged at 12000 rpm at 4℃ for 10 min and then the protein was precipitated at -20℃ for 1 h. The supernatant was then centrifuged at 12000 rpm at 4℃ for 10 min and vacuum-dried. After that, 200μL 50% acetonitrile solution was added for redissolution, and then centrifuged at 14000 rpm at 4℃ for 15 min after vortex. Furthermore, the supernatant was then injected into the UPLC-Orbitrap-MS system (UPLC, Vanquish; MS, HFX).

**2. Preparation of the placebo granules solution**

We weighed 100 mg of the placebo granules and put them into a centrifugation tube, subsequently adding 1 mL extraction solution (water/acetonitrile/isopropyl alcohol, 1:1:1), swirl them for 60 s, and extract them by ultrasound at low temperature for 30 min. The supernatant was centrifuged at 12000 rpm at 4℃ for 10 min and then the protein was precipitated at -20℃ for 1 h. The supernatant was then centrifuged at 12000 rpm at 4℃ for 10 min and vacuum-dried. After that, 200μL 50% acetonitrile solution was added for redissolution, and then centrifuged at 14000 rpm at 4℃ for 15 min after vortex. Furthermore, the supernatant was then injected into tUPLC-Orbitrap-MS system (UPLC, Vanquish; MS, HFX).

**3. Chromatographic and mass spectrometry conditions**

The chromatography was performed on Waters HSS T3 (100×2.1 mm, 1.8 μm) at 40℃. 0.1% formic acid acetonitrile solution (A) and 0.1% formic acid water solution (B) were used for mobile phase analysis with a flow rate of 0.4mL/min. The injection volume was 2μl. The QExactive HFX high resolution mass spectrometry system of Thermo Company in the United States was used to acquire the primary and secondary spectra, equipped with an Electrospray ionization (ESI) source, sheath gas 40 arb, auxiliary gas 10 arb, and auxiliary gas 40 ARB. Ion spray voltage +3000 V/-2800 V, temperature 350℃, ion transfer tube temperature 320℃. The scanning mode is Full-ms-ddMS2, and the scanning mode is positive ion/negative ion. The scanning range of primary mass spectrometry is (scan m/z range): 70-1050 Da, the primary resolution is 70,000, and the secondary resolution is 17500.

**4. Experimental results**

We analyzed the main components of the SHG and placebo granules using UPLC-Q-Exactive HFX-MS. The chemical structures of the main components were qualitatively identified using mass spectrometry software Progenesis QI (Waters Corporation, Milford, USA). Based on MS and MS/MS analysis results and existing reference materials, 14 chemical compounds were identified in the SHG, including PSI-6130, (+)-Catechin hydrate, Homoplantaginin, Dirithromycin, 1,6-Digalloylglucose, Isoforsythiaside, Hexanorcucurbitacin F, Asterbatanoside A,

Rhodiocyanoside A, Zeylenone, 6-0-Vanilloylsucrose, antcin A, Cyclohexanecarboxylic acid, Chlorogenic Acid, and Cryptochlorogenic acid. BPI chromatograms of the SHG in positive ion mode and negative ion mode were shown in Figure 1.


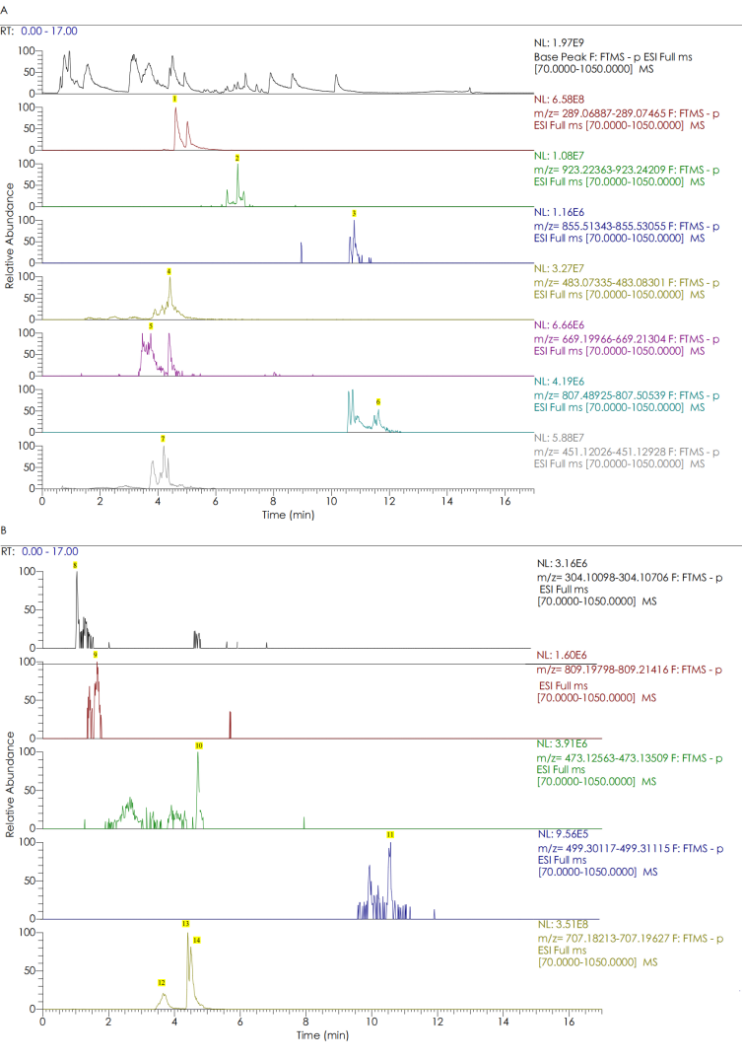


**Figure 1.** Base peak intensity (BPI) chromatogram of SHG in negative ion mode (1: (+)-Catechin hydrate; 2: Homoplantaginin; 3: Dirithromycin; 4: 1,6-Digalloylglucose; 5: Isoforsythiaside; 6: Hexanorcucurbitacin F; 7: Asterbatanoside A; 8: Rhodiocyanoside A; 9: Zeylenone; 10: 6-0-Vanilloylsucrose; 11: antcin A; 12: Cyclohexanecarboxylic acid; 13: Chlorogenic Acid; 14: Cryptochlorogenic acid.)

**
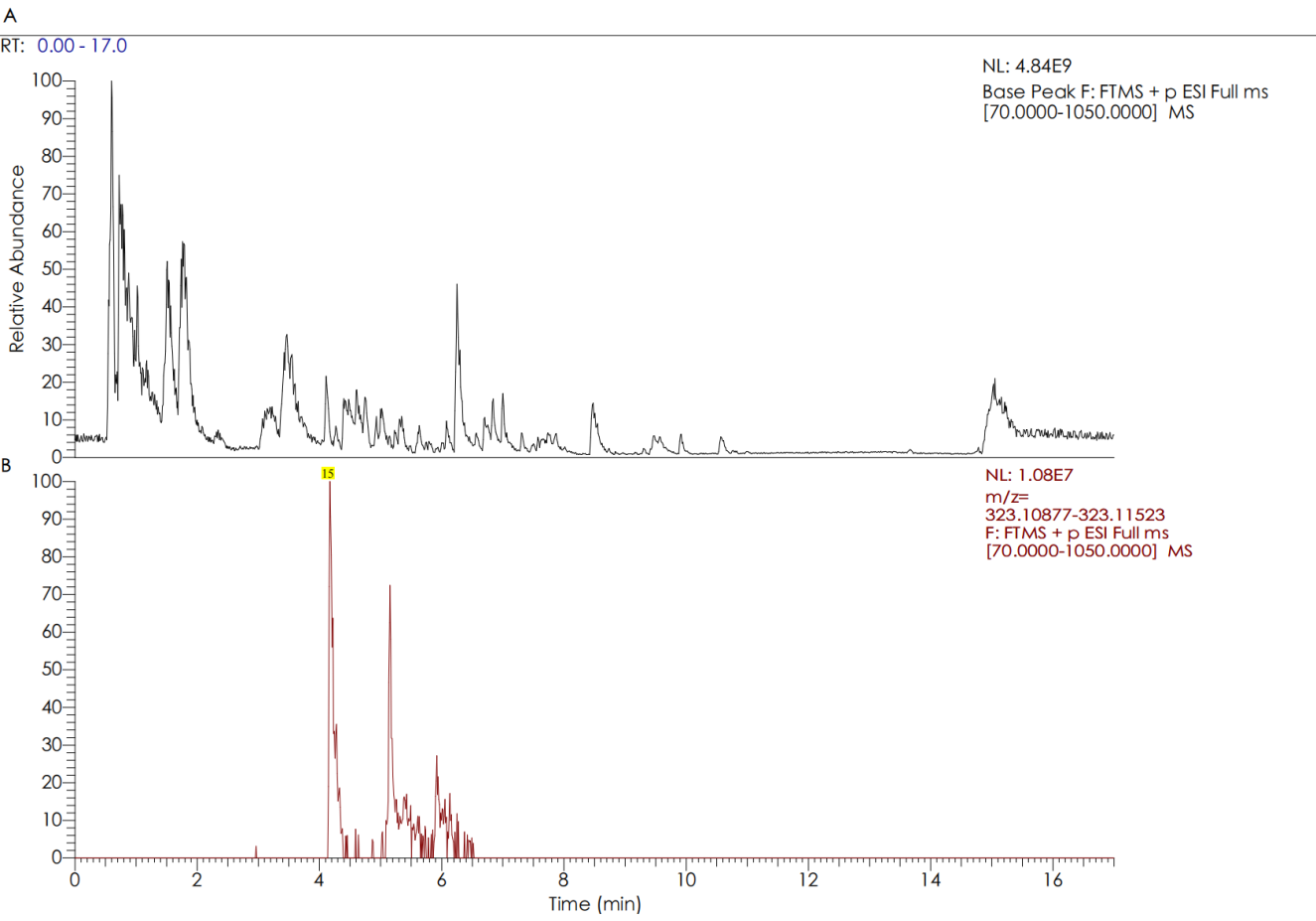
**

**Figure 2.** Base peak intensity (BPI) chromatogram of SHG in postive ion mode (15: PSI-6130.)
